# Intradiscal Injection of Induced Pluripotent Stem Cell-Derived Nucleus Pulposus-Like Cell-Seeded Polymeric Microspheres Promotes Rat Disc Regeneration

**DOI:** 10.1155/2019/6806540

**Published:** 2019-05-02

**Authors:** Kaishun Xia, Jian Zhu, Jianming Hua, Zhe Gong, Chao Yu, Xiaopeng Zhou, Jingkai Wang, Xianpeng Huang, Wei Yu, Liming Li, Jianqing Gao, Qixin Chen, Fangcai Li, Chengzhen Liang

**Affiliations:** ^1^Department of Orthopedics, The Second Affiliated Hospital, Zhejiang University School of Medicine, #88 Jie Fang Road, Hangzhou, 310009 Zhejiang, China; ^2^Orthopedics Research Institute of Zhejiang University, #88 Jie Fang Road, Hangzhou 310009, China; ^3^Department of Radiology, The Second Affiliated Hospital, Zhejiang University School of Medicine, #88 Jie Fang Road, Hangzhou, 310009 Zhejiang, China; ^4^Institute of Pharmaceutics, College of Pharmaceutical Sciences, Zhejiang University, Hangzhou, 310058 Zhejiang, China

## Abstract

**Background:**

Cell replacement therapy is an attractive alternative for treating degenerated intervertebral discs (IVDs), which are related to the reduction of nucleus pulposus-like cells (NP-lCs) and the loss of the extracellular matrix. Induced pluripotent stem cells (iPSCs) which resemble embryonic stem cells are considered to be a potential resource for restoring NP-lCs and disc homeostasis. Here, we proposed an efficient two-step differentiation protocol of human iPSCs into NP-lCs and continuously tested their *in vivo* ability to regenerate IVDs.

**Methods:**

A polymeric gelatin microsphere (GM) was generated for sustained release of growth and differentiation factor-5 (GDF-5) and as a cell delivery vehicle of NP-lCs. By injecting NP-lC-seeded GDF-5-loaded GMs into the rat coccygeal intervertebral discs, the disc height and water content were examined with the molybdenum target radiographic imaging test and magnetic resonance imaging examination. Histology and immunohistochemistry results were shown with H&E, S-O-Fast Green, and immunohistochemistry staining.

**Results:**

We demonstrated that the injection of NP-lC-seeded GDF-5-loaded GMs could reverse IDD in a rat model. The imaging examination indicated that disc height recovered and water content increased. Histology and immunohistochemistry results indicated that the NP cells as well as their extracellular matrix were partially restored.

**Conclusions:**

The results suggest that NP-lC-seeded GDF-5-loaded GMs could partially regenerate degenerated intervertebral discs after transplantation into rat coccygeal intervertebral discs. Our study will help develop a promising method of stem cell-based therapy for IDD.

## 1. Introduction

Low back pain (LBP) affects approximately 632 million individuals globally [1]. Intervertebral disc degeneration (IDD) is considered to be a common initiating factor of LBP [2]. The intervertebral discs (IVDs) are comprised of three structural elements: the annulus fibrosus (AF), the nucleus pulposus (NP), and the end plates (EPs) [3]. NP is a less structured, hydrophilic, proteoglycan-rich gelatinous core that is sandwiched between the two EPs. The proteoglycans are composed primarily of aggrecan (ACAN), collagen type II (COL2), collagen type IV, and elastic fibers [4]. The IVD degeneration leads to a decline of COL2 in the extracellular matrix (ECM), and the reduction of NP cells and the loss of ECM are the main causes of IDD. Replenishing with autologous cells could be an effective way to restore NP cells and ECM and reverse disc degeneration *in vivo* [5].

Induced pluripotent stem cells (iPSCs) are reprogrammed somatic cells which resemble embryonic stem cells and can be differentiated into numerous types of cells [6–9]. Therefore, human iPSCs (hiPSCs) are considered to be a potential resource for restoring NP cells and disc homeostasis. However, current studies on the induction of iPSCs to differentiated NP cells are very limited [10–14]. Numerous studies have demonstrated that the notochord is the precursor organization of NP, which originates from the mesoderm [15]. Previous studies have identified transcription factors and gene regulatory networks involved in IVD formation [16]. Therefore, our first step was to induce iPSCs to differentiate into mesoblastic cells (MCs) and then induce MCs into NP-like cells (NP-lCs), which may be more efficient.

Growth factor therapy is an attractive method for repairing degenerated IVDs [17]. Growth and differentiation factor-5 (GDF-5) is a promising factor that could inhibit IDD in a mouse disc degeneration model [18–22], and it promotes NP-like differentiation of stem cells [23]. Several factors such as bone morphogenetic protein 7 [24] and basic fibroblast growth factor (bFGF) [25] could promote NP cell proliferation and IVD regeneration, but multiple injections of these factors are needed due to a short life *in vivo* [26].

Recently, microparticulate systems have attracted increasing attention in tissue engineering. Gelatin is a biodegradable and biosafety biomaterial widely used in medicine, pharmaceutics, and industry [27]. In this study, we constructed a sustained release system by packaging GDF-5 into gelatin microspheres (GMs). With the degradation of gelatin, GDF-5 will be constantly released into the NP tissue to inhibit IDD and promote notochord cells to differentiate into NP-lCs. In addition, GMs provide a carrier for NP-lCs, which would help the cells to survive in a harsh IVD microenvironment. Next, we applied NP-lC-seeded GDF-5-loaded GMs to reverse degenerated IVDs in a rat model.

## 2. Material and Methods

### 2.1. Reagents and Antibodies

Gelatin was purchased from Nitta Gelatin Inc. (Osaka, Japan). Low molecular weight heparin (LMWH), bovine serum albumin (BSA), and dexamethasone were purchased from Sigma-Aldrich (USA). Recombinant GDF-5, transforming growth factor beta 1 (TGF-*β*1), and bone morphogenetic protein 2 (BMP2) were from PeproTech (Rocky Hill, USA). The antibodies against aggrecan and COL2 were purchased from Abcam (UK). All reagents used were of at least ACS grade.

### 2.2. Preparation of GDF-5-Loaded Gelatin Hydrogel Microspheres

**Figure 1:**
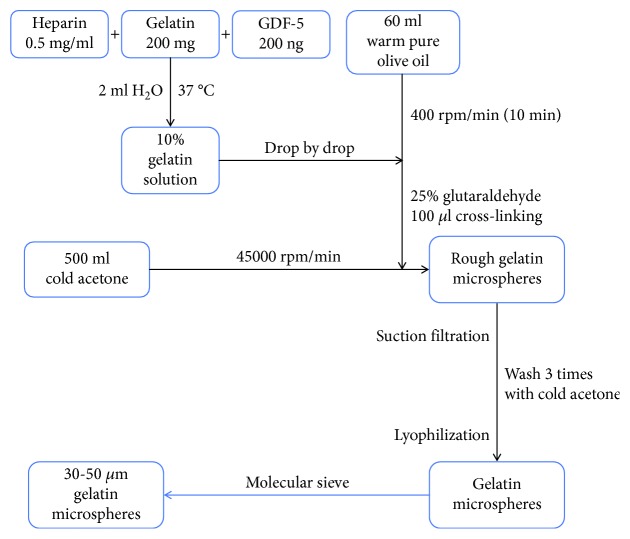
Flow chart of the synthesis of GDF-5-loaded GMs.

Recombinant GDF-5 was used at 100 ng/ml based on previous literature [23]. GDF-5 (100 *μ*g/ml, 2 *μ*l) was premixed with a heparin solution (0.5 mg/ml, 2 ml) and added to 10% (*w*/*v*) gelatin (200 mg) [28], and the mixture was then incubated at 37°C for 30 min. Then, the GMs were prepared by chemical crosslinking in a water-in-oil state. Briefly, the prepared mixture was added dropwise at 37°C into 60 ml of olive oil (pharmaceutical grade, Aladdin, China), with constant stirring at 400 rpm for 10 min to form a water-in-oil emulsion, and 25% glutaraldehyde (100 *μ*l) was added as a chemical crosslinking agent. The noncrosslinked GMs were prepared at 4°C in a refrigerator. Then, the microspheres were washed three times with precooled acetone (4500 rpm, 4°C, 5 min) to preclude residual oil. The microspheres were vacuum freeze-dried overnight and filtered using sieves (apertures 30 and 50 *μ*m). The average diameter of 200 microspheres was measured using ImageJ software (NIH, Bethesda, USA) (Figure 1).

### 2.3. Measurement of the Encapsulation Percentage and Loading Efficiency

Microspheres were prepared as described above with GDF-5 replaced by 2 mg BSA (MtBSA). 10 mg of microspheres (total weight, Mt) was incubated in 5 ml of phosphate-buffered saline (PBS) at 37°C for 30 min until they were dissolved completely. The released BSA concentration (MBSA) was measured by the bicinchoninic acid (BCA) assay. The encapsulation efficiency and loading efficiency were calculated as follows:

(1) $$\begin{array}{c} \text{Encapsulation percentage }\left(\text{EN}\%\right)=\frac{\text{MBSA}}{\text{MtBSA}}*100\%,\\ \text{Loading efficiency }\left(\text{LE}\%\right)=\frac{\text{MBSA}}{\text{Mt}}*100\%. \end{array}$$

### 2.4. Characterization of Microsphere Morphology and *ζ*-Potential Measurement

The microspheres were examined by scanning electron microscopy (SEM; SU8010, Hitachi, Japan) and transmission electron microscopy (TEM; HT7800, Hitachi, Japan). The samples were mounted onto aluminum stubs with a carbon tape and sputter coated with a thin gold film (Emitech, K550) and then observed under an SEM. For TEM, the samples were prefixed with resin and sectioned using an ultramicrotome. The *ζ*-potential (surface charge) of GDF-5-loaded GMs was scanned at 25°C using Zetasizer (Malvern, Worcestershire, UK).

### 2.5. *In Vitro* Release of GDF-5 from GMs

The in vitro release of GDF-5 was evaluated using 100 mg of GMs. Briefly, the microspheres were decentralized in 1 ml PBS and then placed on an orbital shaker. The medium was removed and replenished at different time points, and the solution was frozen and stored at -20°C for the enzyme-linked immunosorbent assay (ELISA) (Human GDF-5 ELISA Kit, CUSABIO, Wuhan, CN).

### 2.6. hiPSC Culture and Differentiation

DYR0100 hiPSCs were kindly provided by the Chinese Academy of Sciences. The cells were cultured on prepared Matrigel (BD Matrigel™ hESC-qualified Matrix, USA) in TeSR™-E8™ Basal Medium (STEMCELL Technologies, Vancouver, Canada). The medium was changed every 2 days, and the cells were harvested after passage for 4-6 days for subsequent experiments. The cultured hiPSCs were first differentiated into MCs, followed by further differentiation into NP-lCs. On day 0, hiPSCs were harvested and seeded into a 6-well plate with TeSR™-E8™, and the medium was replaced daily for 8 days with differentiation medium, which was based on Dulbecco's modified Eagle's medium (DMEM, Gibco, Australia) supplemented with 1.25 mg/ml BSA, 1× ITS, 0.1 *μ*M dexamethasone, 1 mM sodium pyruvate, 0.17 mM ascorbic acid-2-phosphate, and 0.35 mM proline (Table 1, stage 1). After the cells exhibited a transformed morphology, they were cultured in NP differentiation medium (Table 1, stage 2) for 21 days to be differentiated into NP-lCs.

### 2.7. Cell Seeding and Growth on GDF-5-Loaded GMs

1 mg of microspheres and 1 × 10^6^ NP-lCs were incubated in a 15 ml centrifuge tube for 3 days. Then, 1 *μ*l of DiD (10 mg/ml, Invitrogen™, USA) was added into the medium and incubated at 37°C for 30 min, and the solution was then shifted into a 6-well plate and observed by inverted fluorescence microscopy.

### 2.8. Cytotoxicity and Cell Proliferation Assay

The cytotoxicity of GMs was determined after 1, 3, 5, and 7 days of coculture with NP-lCs. The supernatant was collected at each time point, and lactate dehydrogenase (LDH) content was measured by using an LDH assay kit (Jiancheng Bioengineering Institute, China). The absorbance values at 440 nm were used to assess the content of LDH. Cell proliferation was determined after 1, 3, 5, and 7 days of coculture with NP-lCs. At each time point, 200 *μ*l of CCK-8 assay kit solution (Dojindo Molecular Technologies, Japan) was added to replace the culture medium and incubated at 37°C for 4 h. Then, 150 *μ*l of the supernatant was shifted into a 96-well plate, and the absorbance values were measured at 450 nm.

### 2.9. Cytochemical Staining

After 21 days of differentiation, the cells were stained with Alcian blue (Sigma-Aldrich) and Safranin O (S-O) (Sigma-Aldrich). For immunofluorescence detection, the cells were washed with PBS 3 times and fixed using 4% paraformaldehyde, and the cell membrane was disrupted with 0.3% Triton X-100. Then, the cells were incubated with 5% BSA at room temperature (RT) for 30 min and incubated overnight at 4°C with the appropriate primary antibody, COL2 (1 : 400, Abcam) or ACAN (1 : 250, Abcam), followed by incubation with a donkey anti-mouse secondary antibody conjugated to FITC (1 : 200), and the cells were observed under a fluorescence microscope.

### 2.10. PCR

RNA was isolated from the differentiated NP-lCs using TRIzol (Invitrogen). Then, cDNA was generated using a PrimeScript RT reagent kit (TaKaRa Bio, Japan). The mRNA expression level was tested using the StepOnePlus Real-Time PCR System (Applied Biosystems, CA, USA) and SYBR Green (TaKaRa Bio, Japan). The primers were synthesized by Sangon Biotech (Shanghai, China) and are presented in Table 2. 18S rRNA was used as the housekeeping gene. The results were evaluated by the 2-ΔΔCt method.

### 2.11. Animal and Surgical Procedures

Sprague-Dawley rats (150 g) were purchased from the Zhejiang Chinese Medical University Laboratory Animal Research Center. All procedures were approved by the Institutional Animal Care and Use Committee of Zhejiang University. The surgical procedure was described previously [29]. Briefly, 3% pentobarbital sodium was injected intraperitoneally as the anesthetic after coccygeal vertebrae Co 7/Co 8 and Co 8/Co 9 were localized using digital palpation and double confirmed by a trial radiograph. A sterile 20-gauge needle (outer diameter, 0.91 mm) was inserted just through the AF into the middle of the NP [30, 31]. The needle was rotated 360° and held for 30 s. 80 male rats were divided into five groups: the normal control (NC) group (without needle puncture or DMEM injection), the degeneration control (DC) group (needle puncture after 2 weeks and then DMEM injection), the GM group (needle puncture after 2 weeks and then GM injection), the NP-lC group (needle puncture after 2 weeks and then NP-lC injection), and the GMN group (needle puncture after 2 weeks and then injection of GM-loaded NP-lCs).

### 2.12. Disc Height Measurement

After injection for 4, 8, 16, and 24 weeks, caudal disc radiographs were collected by a molybdenum target radiographic imaging unit (GE Mammography DMR Bucky 18 × 24, UK) to detect the disc height after injection. All the anesthetized rats were placed in a supine position to keep their tails straight. The digital images were obtained by a skilled observer who manipulated the computer radiographic plate system (AGFA Diagnostic Center Compact Plus, AGFA HealthCare, Belgium). The disc height index (DHI%) was calculated using the ImageJ software (NIH, Bethesda, USA) by three blinded observers.

### 2.13. MRI Procedure and Data Processing

T2-weighted sections in the sagittal plane and transverse plane were obtained by 3.0-T MRI (GE Medical Systems, UK) to show the water content and the caudal spine structure at 4, 8, 16, and 24 weeks after injection. All the anesthetized rats were placed in a supine position to keep their tails straight. The parameter settings were as follows: echo time, 80 ms; spin echo repetition time, 2275 ms; field of view, 5 cm; number of excitations, 8; slice thickness, 1.5 mm; and no phase wrap. The images were processed using a GE ADW4.2 workstation [32], and NP was evaluated using the MRI index [33]. The disc height was measured by three investigators in an independent and blinded fashion.

### 2.14. Histological Analysis

The rats were euthanized by intraperitoneal injection of pentobarbital sodium (150 mg/kg), and the specimens were washed with ultrapure water, then fixed in 4% paraformaldehyde for 3 days. After decalcification with 10% EDTA for 2 months, the specimens were embedded with paraffin and cross-sectioned (5 *μ*m). The specimens were stained using hematoxylin and eosin (H&E), followed by S-O-Fast Green. The morphology and cellularity of the IVDs were assessed by three investigators blinded and independently using a grading scale [32].

### 2.15. Immunohistochemistry

After deparaffinization in xylene, the specimens were rehydrated and treated with 3% H_2_O_2_ for 15 min. Then, the specimens were blocked with goat serum for 30 min at RT and incubated at 4°C overnight with a mouse monoclonal COL2 antibody (1 : 200, Abcam). After washing with PBS for 3 times, the specimens were incubated at 37°C for 30 min with biotin-labeled rabbit anti-mouse IgG (1 : 200), and staining was detected by the streptavidin-biotin complex (SABC) method.

### 2.16. Statistical Analyses

The data were shown as the mean ± standard deviation (SD), and the statistical significance was evaluated using two-tailed Student's *t*-test. All statistical analyses were performed using SPSS software (version 22.0 for Windows; SPSS Inc., USA). The *p* values were two-tailed, and a *p* value < 0.05 was considered statistically significant.

## 3. Results

### 3.1. Characterization of GDF-5-Loaded GMs

**Figure 2:**
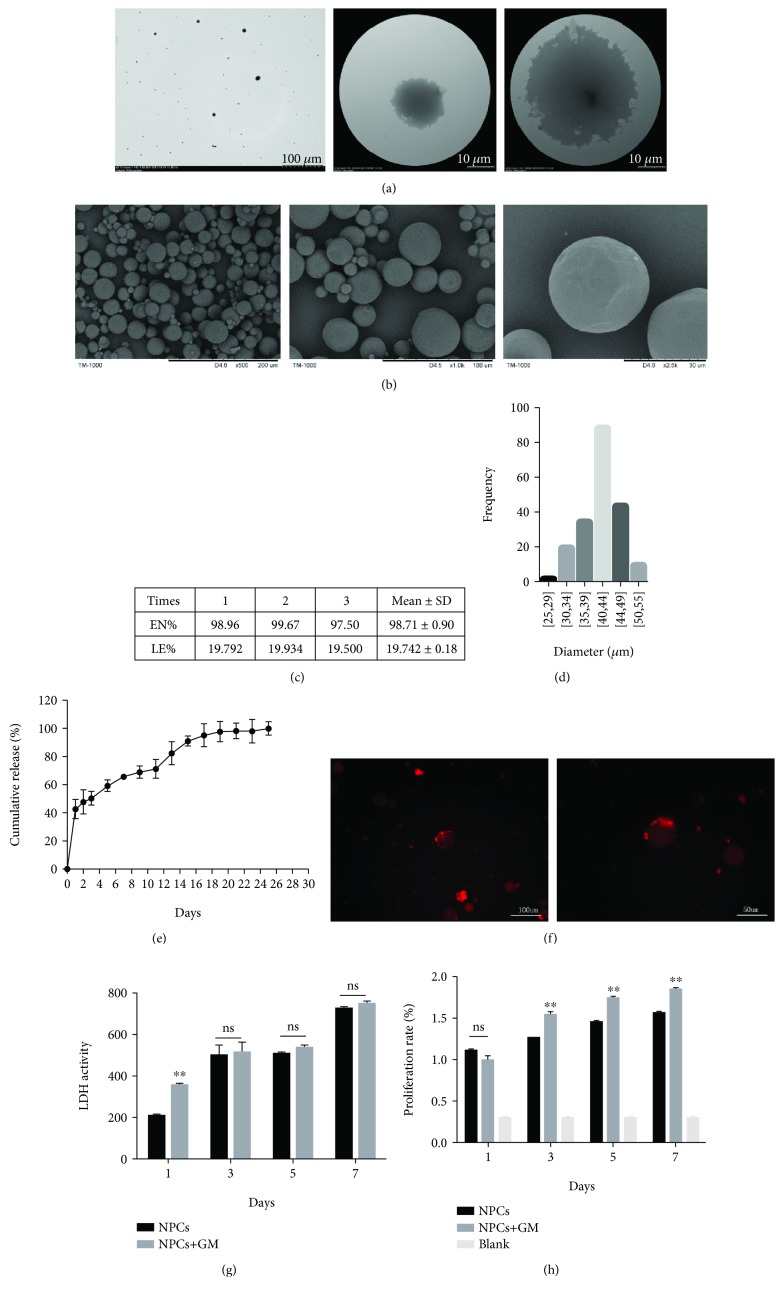
Characterization of the GMs. (a) TEM images of GMs at 700x and 3000x. Scale bar = 100 *μ*m and 10 *μ*m. (b) SEM images of GMs at 500x, 1000x, and 2500x. Scale bar = 200 *μ*m, 100 *μ*m, and 30 *μ*m. (c) The EN% and LE% of different BSA concentrations in the GMs. (d) The average diameter of the GMs. (e) Kinetic curves of the sustained release of GDF-5 from GMs. Data are presented as the mean ± SD. (f) DiD dye showed that hiPSCs adhered to the GMs. Scale bar = 100 *μ*m and 50 *μ*m. (g) The LDH assay for the cytotoxicity of GMs on hiPSCs. (h) The proliferation of hiPSCs cultured with GMs.

SEM showed that the dry GDF-5-loaded GMs had a relatively smooth outer surface (Figure 2(a)). After dissolving GMs in PBS, TEM showed that the microspheres had a slightly rough surface, which could provide a perfect place for NP-lCs to adhere (Figure 2(b)). When BSA was used to replace GDF-5, EN% was 98.71 ± 0.90 and LE% was 19.742 ± 0.18 (Figure 2(c)). The average diameter of GDF-5-loaded GMs was 25-55 *μ*m, with a Gaussian distribution (Figure 2(d)). In addition, the *ζ*-potential of −5.32 ± 0.35 indicated that the microspheres had a slight negative charge and were approximately electroneutral. In addition, the kinetic curves of LMWH-bound GDF-5 (100 ng) demonstrated the sustained release of GDF-5 from GDF-5-loaded GMs (Figure 2(e)).

### 3.2. The Effectiveness of GDF-5-Loaded GMs for the Delivery of NP-lCs

DiD is a dye delivery solution that can be added into culture medium with no effect on cell proliferation, and the results showed that NP-lCs adhered to GMs (Figure 2(f)), which indicated that GMs could load NP-lCs to form a cell delivery system. The LDH assay indicated that GDF-5-loaded GMs had no cytotoxicity on NP-lCs (Figure 2(g)). In addition, GDF-5-loaded GMs increased slightly the proliferation of NP-lCs (Figure 2(h)).

### 3.3. The Differentiation of hiPSCs into NP-lCs

**Figure 3:**
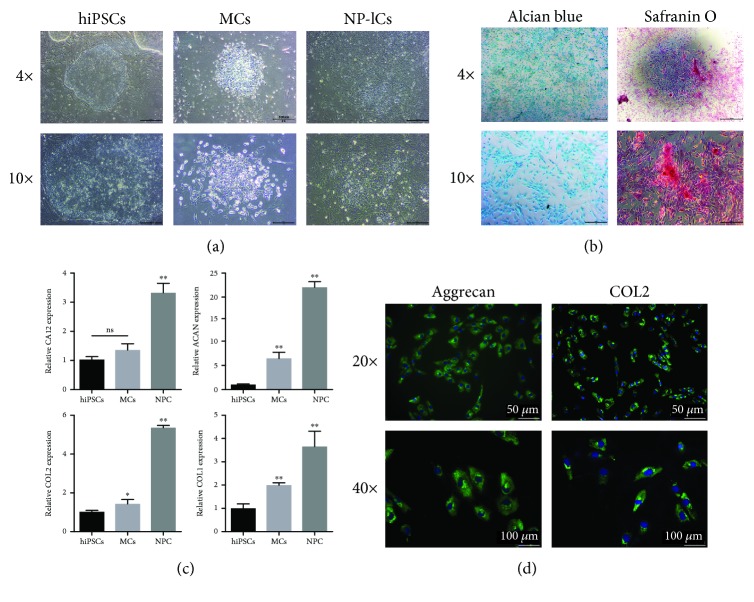
The differentiation of hiPSCs into NP-lCs. (a) Optical microscopy images of the differentiation process: hiPSCs (I and II), MCs (III and IV), and NP-lCs (V and VI). 4x (scale bar = 500 *μ*m), 10x (scale bar = 200 *μ*m). (c) Alcian blue staining and S-O staining of the differentiated NP-lCs. 4x (scale bar = 500 *μ*m), 10x (scale bar = 200 *μ*m). (d) qRT-PCR analysis of COL2, ACAN, CA12, and COL1 mRNA expression levels in NP-lCs, MCs, and hiPSCs; ^∗∗^*p* < 0.001. (e) Immunofluorescence images of COL2 and ACAN staining in NP-lCs. 10x (scale bar = 100 *μ*m), 20x (scale bar = 50 *μ*m).

With an optical microscope, we observed the morphology at each differentiation stage of hiPSCs (Figure 3(a)). After differentiation into NP-lCs, Alcian blue staining and S-O staining demonstrated that the cellular proteoglycan ECM contained specific components of cartilage tissues (Figure 3(b)). Moreover, mRNA expression levels of COL2, ACAN, carbonic anhydrase XII (CA12), and collagen type I (COL1) were significantly higher in the differentiated group than in the hiPSC group (Figure 3(c)). In addition, immunofluorescence results showed that the differentiated NP-lCs were positive for Col2 and ACAN (Figure 3(d)).

### 3.4. Radiographic and MRI Assessment of Discs in Each Group

**Figure 4:**
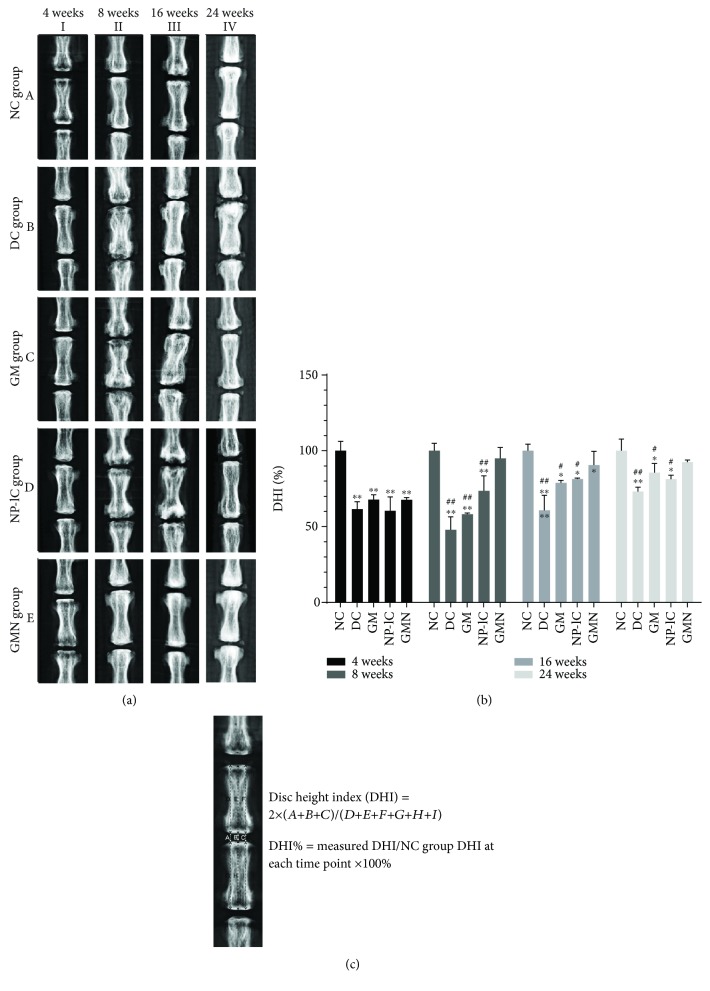
Radiographs results. (a) Radiographs of all the five groups, which were obtained at 4, 8, 16, and 24 weeks after injection. (b) DHI% was calculated from digitized radiographs using ImageJ. (c) The disc height changes. ^∗∗^*p* < 0.001, ^∗^*p* < 0.05 vs. the NC group; ^##^*p* < 0.001, ^#^*p* < 0.05 vs. the GMN group.

Figure 4 shows the DHI% values of different groups at 4 time points. The values were significantly lower at each time point in the DC, GM, and NP-lC groups than in the NC group, and the GMN group had significantly higher DHI values than the DC, GM, and NP-lC groups at weeks 8, 16, and 24.

**Figure 5:**
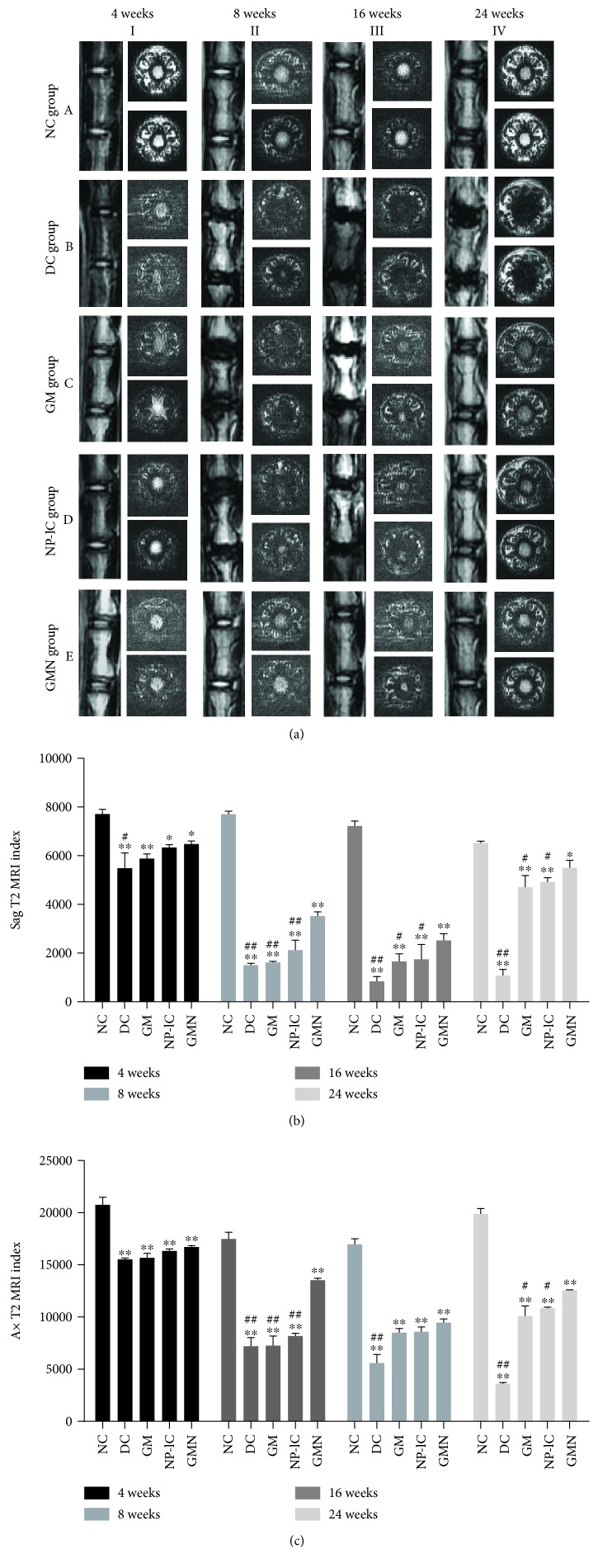
MRI results. (a) Representative T2 MRI scans. (b) The sagittal plane T2 MRI index of each group. (c) The transverse plane T2 MRI indexes of different groups. ^∗∗^*p* < 0.001, ^∗^*p* < 0.05 vs. the NC group; ^##^*p* < 0.001, ^#^*p* < 0.05 vs. the GMN group.

Figure 5 reveals the NP tissue structure and water content by MRI results. The Sag T2 sequence showed the sagittal plane of the disc, while the Ax T2 sequence showed the transverse plane. At each time point, the index was higher in the NC group than in other groups in both Sag and Ax sequences. In addition, in the Sag sequence, the GMN group showed a significantly higher index than the DC, GM, and NP-lC groups at weeks 8, 16, and 24. In the Ax sequence, the GMN group had a significantly higher index than the DC, GM, and NP-lC groups at weeks 8 and 24.

### 3.5. Histological and Immunohistochemical Analysis of Discs in Each Group

**Figure 6:**
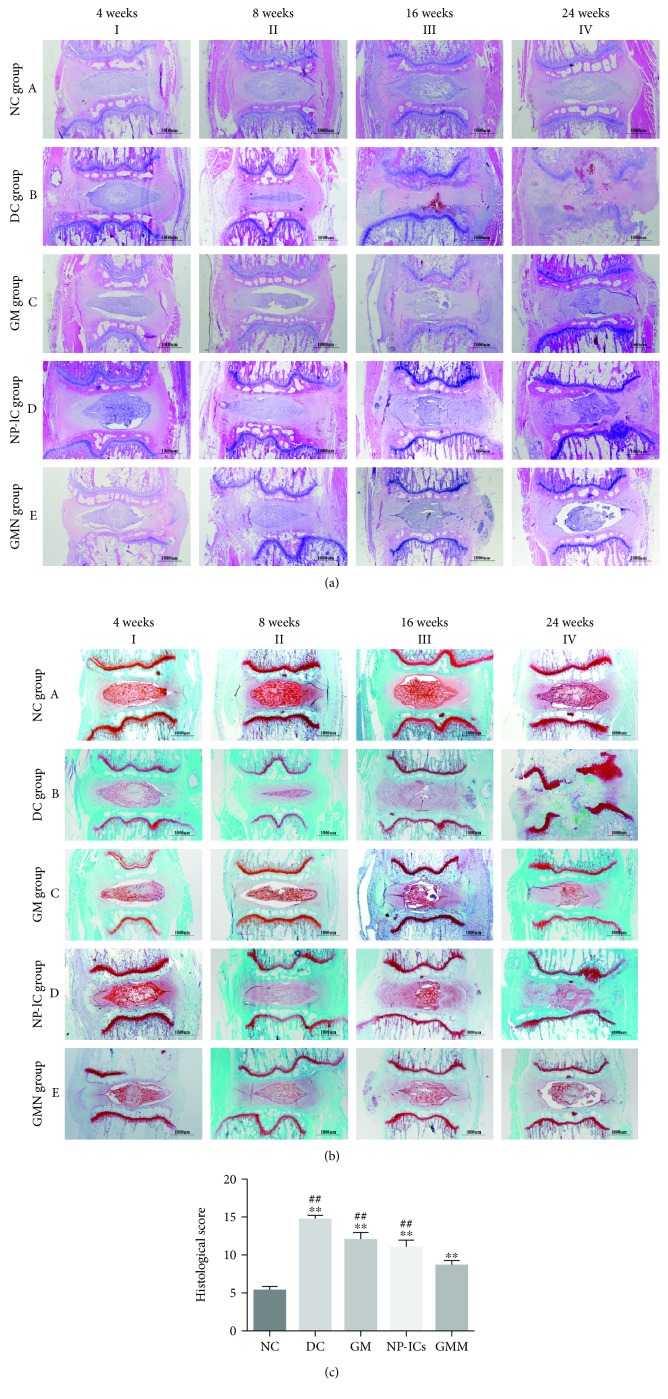
H&E and S-O staining of the disc samples. (a) Representative H&E staining of disc samples from different groups at 4, 8, 16, and 24 weeks after injection (scale bar = 1,000 *μ*m). (b) Representative S-O staining of disc samples from different groups at 4, 8, 16, and 24 weeks after injection (scale bar = 1,000 *μ*m). (c) Histological scores of the five groups at 24 weeks postinjection. ^∗∗^*p* < 0.001, ^∗^*p* < 0.05 vs. the NC group; ^##^*p* < 0.001, ^#^*p* < 0.05 vs. the GMN group.

The discs were categorized into 3 levels, normal, moderately degenerated, and severely degenerated, according to the histological grading scale. H&E and S-O staining showed that at week 24 the NC group presented a mixture of small chondrocyte-like cells and larger vacuolated cells; the AF was well organized with lamellar sheets of collagen. In contrast, NP and AF were disorganized in the DC group, and there was gradual degeneration of the NP tissue. The AF of the GM and NP-lC groups was narrowed and distorted, and the injured regions were filled with fibrous connective tissues. However, in the GMN group, the distribution of the cells and ECM and the order of AF were regular compared to those in the DC, GM, and NP-lC groups (Figures 6(a) and 6(b)). The histological scores were significantly higher in the DC, GM, NP-lC, and GMN groups than in the NC group, while the GMN group had a significantly lower score than the DC, GM, and NP-lC groups (Figure 6(c)).

**Figure 7:**
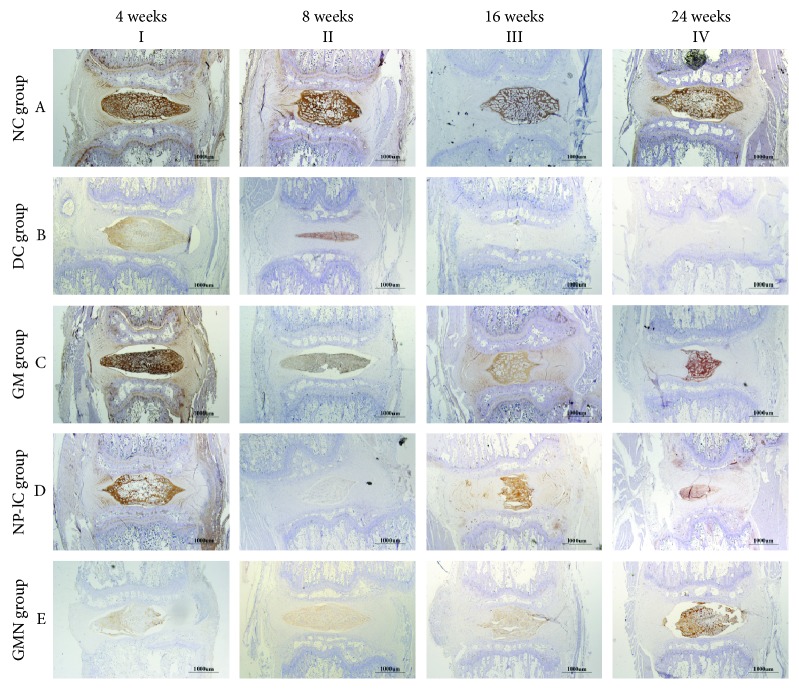
Immunohistochemistry staining of COL2 in disc samples from different experimental groups. Scale bar = 1,000 *μ*m.

The distribution of COL2 staining in the discs of the NC group was much the same as that of S-O staining. In the DC group, the time gradient showed a gradually fading change, as the IVB was negative for COL2. In the GM and NP-lC groups, COL2 expression in the shrinking NP area was not as strong as that in the NC group. In contrast, the GMN group showed stronger expression of COL2 at week 24 than at weeks 8 and 16. However, the GM group (IVC) exhibited stronger expression of COL2 than the GMN group at week 24 (Figure 7).

## 4. Discussion

IDD is an age-related chronic process, and the reduction of NP cells and the destruction of ECM seemed to be the initiating factors that result in IDD and subsequent LBP [34, 35]. Stem cell transplantation was considered a promising method for increasing ECM and restoring NP cells [36]. iPSCs have shown the potential to act as a provider of NP cells in vitro [10–14]. Therefore, this study was aimed at exploring the in vivo ability of iPSCs to regenerate degenerated IVDs.

GMs are considered to be a suitable biomaterial in stem cell-based therapy [37], and their degradation and biosafety have been widely tested [28]. Based on the literature [18–23], we chose GDF-5, which could promote NP differentiation and inhibit IDD, and added it into the delivery system. The combination of GDF-5 with GMs turned out to become a controlled release system and could maintain the concentration of growth factors to avoid their short life in vivo. Our in vitro study indicated that GDF-5-loaded GMs had no cytotoxicity, promoted cell proliferation, and achieve the sustained release of GDF-5.

In this study, we developed a two-step directed differentiation protocol for hiPSCs. Stage 1 (days 1-8) aimed at differentiating hiPSCs into the MCs and stage 2 (days 9-30) were to induce the MCs into NP-lCs. To test the *in vitro* differentiation ability of hiPSCs into NP-lCs, we performed Alcian blue and S-O staining and both showed positive results. In addition, Col1, Col2, ACAN, and CA12 expression levels were higher in differentiated cells than in hiPSCs, and the differentiated NP-lCs were immunopositive for Col2 and ACAN. These data indicated that we successfully induced hiPSCs into NP-lCs.

For in vivo experiments, the DC group showed a characteristic degenerated IVD, including decreases in water content and disc height, and a disordered NP structure. The GM group, which was injected with GMs alone, exhibited slightly increased water content and disc height. This may be that GMs can continuously release GDF-5 which stimulated notochord cells to produce ECM [18–22]. However, considering the short half-life of growth factors, the GM group lacked the ability to regenerate IVDs. The inherent avascularity, isolation, and low metabolic activity of the IVDs may explain the poor function and viability of the transplanted stem cells. The NP-lC group, which was injected only with NP-lCs, showed no obvious repair effect. In contrast, in the GMN group in which GMs not only released GDF-5 but also provided adherence support for NP-lCs, the histological score showed a substantial decrease after 24 weeks compared with the DC, GM, and NP-lC groups, indicating that the NP was recovered with increased disc height, water content, and ECM and collagen II synthesis, as well as ordered IVD structure.

Our experimental animal model involved pushing a needle through the AF into the NP tissue, which is an efficient approach to simulate the development of degenerated IVDs, as the degenerated disc will slowly lose its matrix and exhibit reduced height [38, 39]. Several clinical studies have shown that mesenchymal stem cells (MSCs) only partially alleviated the symptoms of IDD after transplantation, with a significantly elevated water content but a lack of disc height recovery [40, 41]. According to embryology, NP cells originate from the mesoderm [42]; thus, we aimed to differentiate iPSCs into NP-lCs, and injecting NP-lCs to restore the NP cell number might be a suitable approach for NP regeneration. Our results showed that the injection of NP-lC-seeded GDF-5-loaded GMs could reverse IDD after transplantation in vivo, with the disc height recovered, the water content elevated, and the NP cells and ECM restored.

## 5. Conclusion

We constructed a biomaterial that could achieve the sustained release of the growth factor GDF-5 and provide the vehicle for the delivery of NP cells. Our in vivo results demonstrate that the injection of NP-lC-seeded GDF-5-loaded GMs could partially regenerate degenerated IVDs after transplantation into rat coccygeal IVDs. Our study will help develop a promising method of stem cell-based therapy for IDD.

## Figures and Tables

**Table 1:** Protocol for stages 1-3 of the differentiation regime for hiPSCs.

Growth factors		WNT3A (ng/ml)	Activin-A (ng/ml)	FGF2 (ng/ml)	NT-4 (ng/ml)	Follistatin (ng/ml)	BMP4 (ng/ml)	TGF-*β*1 (ng/ml)	GDF-5 (ng/ml)	BMP2 (ng/ml)
Stage	Day									
1	1	25	50							
1	2	25	25	20						
1	3	25	10	20			40			
1	4			20	2	100	40			
1	5			20	2	100	40			
1	6			20	2	100	40			
1	7			20	2	100	40			
1	8			20	2	100	40			
2	9~30			20				10	100	20
3	31	Cultures terminated								

**Table 2:** Primers used in quantitative RT-PCR.

Gene	Forward primer (5′ to 3′)	Reverse primer (5′ to 3′)
18S	GAATTCCCAGTAAGTAAGTGCGGGTCATA	CGAGGGCCTCACTAAACCATC
CA12	CGTGCTCCTGCTGGTGATCT	AGTCCACTTGGAACCGTTCACT
ACAN	TGAGGAGGGCTGGAACAAGTACC	GGAGGTGGTAATTGCAGGGAACA
COL1	GTGTTGTGCGATGACGTGATCT	GGTGACTCTGAGCCGTCGG
COL2	TTTCCCAGGTCAAGATGGTC	TCACCTGGTTTTCCACCTTC

## Data Availability

The datasets used and/or analyzed during the current study are available from the corresponding author on reasonable request.

## Abbreviations

IVDs: Intervertebral discs
NP-lCs: Nucleus pulposus-like cells
iPSCs: Induced pluripotent stem cells
MCs: Mesoblastic cells
GM: Gelatin microsphere
GDF-5: Growth and differentiation factor-5
LBP: Low back pain
IDD: Intervertebral disc degeneration
AF: Annulus fibrosus
NP: Nucleus pulposus
EPs: End plates
ACAN: Aggrecan
COL2: Collagen type II
ECM: Extracellular matrix
hiPSCs: Human induced pluripotent stem cells
bFGF: Basic fibroblast growth factor
LMWH: Low molecular weight heparin
BSA: Bovine serum albumin
TGF-*β*1: Transforming growth factor beta 1
BMP2: Bone morphogenetic protein 2
EN: Encapsulation percentage
LE: Loading efficiency
SEM: Scanning electron microscopy
TEM: Transmission electron microscopy
ELISA: Enzyme-linked immunosorbent assay
DMEM: Dulbecco's modified Eagle's medium
LDH: Lactate dehydrogenase
S-O: Safranin O
DHI%: Disc height index
Co: Coccygeal vertebrae
SD: Standard deviation
RT: Room temperature.
